# Adverse Drug Reactions in the Emergency Department: Is There a Role for Pharmacogenomic Profiles at Risk?—Results from the ADRED Study

**DOI:** 10.3390/jcm9061801

**Published:** 2020-06-09

**Authors:** Katja S. Just, Harald Dormann, Marlen Schurig, Miriam Böhme, Jochen Fracowiak, Michael Steffens, Catharina Scholl, Thomas Seufferlein, Ingo Gräff, Matthias Schwab, Julia C. Stingl

**Affiliations:** 1 Institute of Clinical Pharmacology, University Hospital of RWTH Aachen, 52074 Aachen, Germany; kjust@ukaachen.de; 2 Central Emergency Department, Hospital Fürth, 90766 Fürth, Germany; Harald.Dormann@klinikum-fuerth.de; 3 Research Department, Federal Institute for Drugs and Medical Devices, 53175 Bonn, Germany; marlen.schurig@bfarm.de (M.S.); Miriam.boehme@bfarm.de (M.B.); jochen.fracowiak@bfarm-research.de (J.F.); Michael.steffens@bfarm.de (M.S.); Catharina.scholl@bfarm-research.de (C.S.); 4 Internal Medicine Emergency Department, Ulm University Medical Centre, 89081 Ulm, Germany; Thomas.Seufferlein@uniklinik-ulm.de; 5 Interdisciplinary Emergency Department (INZ), University Hospital of Bonn, 53127 Bonn, Germany; ingo.graeff@ukb.uni-bonn.de; 6 Dr. Margarete Fischer-Bosch-Institute of Clinical Pharmacology, 70376 Stuttgart, Germany; Matthias.Schwab@ikp-stuttgart.de; 7 Department of Clinical Pharmacology, University of Tuebingen, 72076 Tuebingen, Germany; 8 Department of Pharmacy and Biochemistry, University of Tuebingen, 72076 Tuebingen, Germany

**Keywords:** adverse drug reactions, pharmacogenomics, clopidogrel, CYP2C19, phenprocoumon

## Abstract

Individual differences in required drug dosages exist based on the pharmacogenomic (PGx) profiles. This study aimed to assess associations between PGx profiles and adverse drug reactions (ADR) that lead to admissions to the emergency department (ED). ADR cases of the prospective multi-center observational trial in EDs (ADRED study) were analyzed (*n* = 776) together with the relevant PGx phenotypes of the enzymes CYP2D6, CYP2C19, CYP2C9, and VKORC1. Overall, the allele frequency distribution in this cohort did not differ from the population frequencies. We compared the frequencies of phenotypes in the subgroups with the drugs suspected of certain ADR, in the remaining cases. The frequency distribution of CYP2C19 differed for the ADR bleeding cases suspected of clopidogrel (*p* = 0.020). In a logistic regression analysis, higher CYP2C19 activity (OR (95% CI): 4.97 (1.73−14.27)), together with age (1.05 (1.02−1.08)), showed an impact on the clopidogrel-suspecting ADRs, when adjusting for the clinical parameters. There was a trend for an association of phenprocoumon-risk profiles (low VKORC1 or CYP2C9 activity) with phenprocoumon-suspecting ADRs (*p* = 0.052). The PGx impact on serious ADRs might be highest in drugs that cannot be easily monitored or those that do not provoke mild ADR symptoms very quickly. Therefore, patients that require the intake of those drugs with PGx variability such as clopidogrel, might benefit from PGx testing.

## 1. Introduction

Adverse drug reactions (ADRs) are common phenomena that impact our health system. Around 6.5% of all consultations in the hospital emergency department (ED) are caused by serious ADRs [1,2,3] that mainly affect older, multi-medicated patients [4]. The majority of ADRs is expected to be dose-related, thereby underlining the importance of choosing the right dose for a patient [5].

Pharmacogenomics (PGx) is the study of the inherited variability affecting individuals’ responses to drug treatment [6]. PGx was described to smoothen the way for personalized medicine, improving the efficacy and safety of drug treatment [7], as well as offering a probable cost-effective solution by preventing serious ADRs [8]. In systematic reviews, those drugs that commonly cause ADRs are shown to be metabolized by polymorphic enzymes [9], which might have a clinical impact on required dosages. Especially, the pharmacogenomic variance in the phase-I enzymes of the cytochrome P450 (CYP) system, which reduces or increases the rate of drug metabolism, is of clinical importance for choosing the right drug dosage [6]. Effective drug dosages can vary up to 10-fold between patients, based on individual pharmacogenomic profiles [10]. Therefore, individuals might be easily overdosed with standard drug dosages, potentially leading to ADRs. Often affected is the metabolism of drugs acting on the central nervous system, such as antidepressants or antipsychotics. Interestingly, those substance classes might play a role for ADRs presenting to the ED [11]. However, a lack of clinical trials that aim to demonstrate the advantages associated with pharmacogenomic testing was often pointed out [12].

So far, the evidence for a clinical PGx effect on ADRs is probably best for non-dose-related hypersensitivity reactions, such as carbamazepine induced Stevens-Johnson syndrome in the carriers of the HLA-B*1502 allele [13], or abacavir-related hypersensitivity in the carriers of the HLA-B*5701 allele [14]. However, there is also increasing evidence that the clinical outcome of patients is better when pharmacogenomic testing results in treatment modifications and adjustments of dosages, which can reduce ED visits and hospitalizations [15]. Apart from this, many dosing guidelines exist for recommending dose modifications in special PGx phenotypes, such as for the vitamin K antagonist warfarin, or the opioid codeine [16,17]. Currently, a large European prospective randomized controlled trial is testing the hypothesis to prevent ADRs with preemptive pharmacogenomic testing [18].

We aimed to assess the importance of pharmacogenomic profiles with regards to the occurrence of serious ADR, in the cases with pharmacogenetic biosamples from our ADRED study.

### 1.1. Study Population

A subset of the multi-center observational study on ‘Adverse Drug Reactions in Emergency Departments’ (ADRED; trial registration: DRKS-ID: DRKS00008979) was analyzed. In brief, the ADRED study collects cases of ADRs in four large emergency departments of tertiary care and academic teaching hospitals in Germany, thereby, presenting 6.5% of all ED admissions [1]. More information on study design and enrolment is published elsewhere [1,11].

Inclusion criteria were adult patients, presenting with symptoms, which according to the WHO-Uppsala Monitoring Centre (UMC) system for causality assessment were seen in a possible, probable, or certain relation to a drug (definition of an ADR). This causality assessment was done by study personnel that consisted of trained physicians and pharmacists, respectively. All patients agreed to participate in the study and provided written informed consent. All cases derived from the first funding phase of the ADRED study (from December 2015 to December 2018) with available biosample were included in the analyses. The study was approved by the responsible ethical committee of the University of Bonn (202/15; 3 November 2015).

### 1.2. Data Collection

Demographical and clinical data were collected. The current drug intake was documented, and a causality assessment on each drug taken was performed by our study personnel. All symptoms that were seen in the context of the ADR were documented on ED arrival. Symptoms were classified according to the medical terminology for drug regulatory authorities (MedDRA) [19] and were documented as low-level terms (LLT). According to MedDRA, every LLT was connected to a higher-level preferred term (PT), which was again connected to the highest level, to system organ classes (SOC). We analyzed symptoms on the PT level, according to the regulatory approach. Comorbidities as diagnoses and admission diagnoses were coded according to the International Classification of Diseases (ICD) version 10 [20]. Diagnoses were evaluated at the second level of the ICD-10 coding system.

Patients’ glomerular filtration rate (GFR) was calculated, based on the serum creatinine level at the time of ED admission, using the formula of the Chronic Kidney Disease Epidemiology Collaboration (CKD-EPI) for those patients with available information of serum creatinine on ED presentation [21]. Therefore, the patients with missing information about ethnicity were assumed as being white, as this was the majority in our population.

### 1.3. Laboratory Methods and Assessment of Metabolic Profiles

Upon enrollment, biosamples which consisted of either blood or buccal swab were collected from each participant who agreed and consented to the study. DNA extraction was done directly after receipt of the samples in the research department of the German Federal Institute for Drugs and Medical Devices. Genomic DNA was isolated using magnetic beads with the MagNA Pure LC DNA Isolation Kit—Large Volume (Roche Diagnostics GmbH., Mannheim, Germany).

Pharmacogenomic testing was carried out using Agena Bioscience’s MassARRAY technology. Genotyping of the pharmacogenes was done using the iPLEX^®^ PGx 74 Panel, together with the VeriDose™ CYP2D6 CNV Panel (both Agena Bioscience, Inc., San Diego, CA, USA). The latter assay detects copy number variations (CNVs) for CYP2D6, even in the presence of non-functional hybrid alleles, including *36, *13, and *68. Based on the called genotype haplotypes, respective star-alleles were automatically generated from the single markers, according to the Clinical Pharmacogenetics Implementation Consortium (CPIC) star-allele nomenclature. Haplotype determination was always performed, even if a single marker was missing for a gene. The individual haplotypes, in turn, form diplotypes that can be directly translated to metabolic profiles, either by looking up in the translation tables of the Human Cytochrome P450 Allele Nomenclature Database, if available, or according to the literature. In the study presented, we assessed phenotypes extrapolated from the genotypes of CYP2D6, CYP2C9, CYP2C19, and VKORC1, based on involvement in the metabolism of the most frequent suspected drugs that caused ADRs in our sample. Diplotypes for CYP2D6 were interpreted by taking the copy number and the hybrid alleles into account, in order to assess the number of functional alleles and to determine the activity score. Based on the activity score, diplotypes were classified into metabolic phenotype groups, according to a recent consensus recommendation by CPIC and the Dutch Pharmacogenetics Working Group [22].

### 1.4. Statistical Analysis

All continuous variables were tested for normal distribution using the Kolmogorov–Smirnov test. As all continuous variables were not normally distributed, population characteristics were described by the medians and interquartile ranges for continuous variables. The categorical variables were shown in absolute numbers and percentages. We assessed the five most frequent admission diagnoses as ICD codes and symptoms at a PT level, respectively. Further, we determined the ten drug substances most often suspected to cause the ADR. For each of the suspected drugs assessed, we calculated the frequencies and analyzed the most common admission diagnoses and symptoms and their respective frequencies.

We calculated frequencies of metabolic profiles of relevant polymorphic pharmacogenes that alter dose requirements (CYP2D6, CYP2C9, CYP2C19, and VKORC1) and compared their frequency distribution with the distribution in the European population, according to the freely available frequency tables on the website of PharmGKB, based on the work of the Clinical Pharmacogenetics Implementation Consortium (CPIC^®^) [23]. The tables that gave the relative frequencies of phenotypes were assessed in May 2020. Thus, we calculated the significance of differences using the browser-based tool QuickCalc of the GraphPad website, for comparing the observed and expected frequency distribution using chi-square test [24].

Furthermore, metabolic profiles were compared with the initial triage in the ED, the seriousness of the ADR on ED arrival and the condition at discharge, respectively. In the next step, all frequent admission diagnoses and symptoms of ADRs were compared with the metabolic phenotypes. Finally, we compared the frequency of the metabolic phenotypes with the frequency of often-suspected substances, with the known pharmacogenomic variance. Thereby, we assessed the ten most frequent suspected substances for their metabolic pathways.

We defined a drug as a substrate of a polymorphic pharmacogene, according to high evidence information, such as available clinical guidelines on the database of PharmGKB [25], and compared the frequency distribution of the respective metabolic phenotypes with the suspicion of this drug to cause ADR.

To assess the influence of metabolic phenotypes on the frequent given anticoagulant phenprocoumon, we combined the CYP2C9 and VKORC1 results in an anticoagulant activity risk profile. Patients that were CYP2C9 normal metabolizer (NM) and VKORC1 normal clotter (NC) were defined as low risk. Patients with either CYP2C9 IM or PM status or VKORC1 intermediate clotter (IC) or poor clotter (PC) were defined as intermediate risk. Patients with low activity of CYP2C9 (intermediate metabolizer (IM) or poor metabolizer (PM)) and low activity of VKORC1 (IC or PC) were defined as high-risk patients for anticoagulant activity.

For each comparison, we used a chi-square test to assess statistically significant differences for the phenotype distribution and a Mantel–Haenszel test testing for a linear trend in the anticoagulant activity risk-profile. A z-test calculated significance levels between single phenotype groups, adjusting for multiple testing using Bonferroni correction. For the suspected substances with a significant impact of the pharmacogenomic profile, we further conducted logistic binary regression analyses. Therefore, we transformed the phenotype in a binary parameter. Those phenotypes that showed a reduced enzyme activity (IM and PM) were combined, and likewise those, with normal and high enzyme activity (normal metabolizer (NM), rapid metabolizer (RM), and ultra-rapid metabolizer (UM)) were summarized. This parameter was presented as CYP_sum_. Likewise, the anticoagulant activity profile was summarized by combining high and intermediate risk versus low risk. We calculated two logistic regression models, including the summarized parameter, first, adjusting for age and sex, and second, adding the number of comorbidities and the number of all drugs taken to adjust for clinical multi-morbidity. Thereby, we calculated the odds ratios (OR) and the corresponding 95% confidence intervals (CI). The statistical analyses were conducted with the IBM^®^ SPSS^®^Statistics (Version 25, IBM Inc., Armonk, NY, USA).

## 2. Results

In total, 776 patients were presented to one of the four EDs due to an ADR and provided biomaterial for genotyping. All genotyped markers included in the iPLEX^®^ PGx 74 Panel to derive the metabolic profiles of the genes CYP2C9 (#10 markers), CYP2C19 (#8), VKORC1 (#1) were in a Hardy–Weinberg Equilibrium (HWE). Derivation of the metabolic profiles for CYP2D6 were also based on all available markers (#15) in the panel, although four markers (rs16947, rs28371725, rs3892097, and rs5030655) showed significant (*p* < 0.05) deviations from HWE in our sample. The overall call rate after quality control was 91.2%. A list of all corresponding diplotypes of the genes CYP2D6, CYP2C9, CYP2C19, and VKORC1, together with their respective phenotypes can be found in the supplement (Table S1).

The 776 patients were mainly older (median 70 years), multi-morbid (median 6 diseases), multi-medicated (median intake of 8 concomitant drugs), and presented with mostly serious and urgent ADRs to the ED. Characteristics of the study population are presented in Table 1.

Patients were admitted to the ED with a median of two different admission diagnoses. In general, 256 different admission diagnoses were given to our study population on ED admission. A large part of the patients were admitted due to a drug-associated gastrointestinal bleeding presented by the most frequent admission diagnosis (17.3% K92: Other diseases of the digestive system—melena and hematemesis) and other bleeding events, such as acute post-hemorrhagic anemia (7.3% D62). Patients presented with a symptom complex of a median of three different symptoms forming the clinical appearance of the ADR. In total, patients complained about 357 different symptoms on ED arrival. The most common symptom was a rather unspecific general physical health deterioration that was seen in 22.9% of our study population.

In total, 324 different drugs were suspected for causing the symptoms leading to ED presentation in our cohort. Antithrombotic agents were prominent in those suspected drugs that presented half of the ten most frequently suspected drugs. More precise information on the most common admission diagnosis and symptom per patients with suspected drug can be found in Table 2. Table 2 shows the 10 most frequently suspected drugs for causing the ADR, leading to ED presentation and their respective admission diagnoses on an ICD level 2, and symptoms on a PT level per patient with suspected drug use (Table 2).

The ten drugs most often suspected for causing the ADR on ED presentation led mainly to bleeding events, such as gastrointestinal bleedings. Of the ten substances, we further analyzed those that were known to be affected by the polymorphic enzyme metabolism to a significant degree (major enzyme involvement). Five substances used for further analyses were—phenprocoumon and the effect of the CYP2C9 and VKORC1 phenotypes, clopidogrel and the effect of the CYP2C19 phenotype, metoprolol and bisoprolol and the effect of the CYP2D6 phenotype, and finally ibuprofen and the effect of the CYP2C9 phenotype.

In the overall study population, the frequency distribution of phenotypes did not differ significantly from the phenotype distribution of the European population given in the CPIC frequency tables provided by PharmGKB.

We tested whether the frequency in the metabolic profiles differed in the subgroups of clinical conditions, such as the triage group, seriousness on ED arrival, or condition at discharge. No difference in phenotype frequency was detected in any of these conditions. Comparisons of triage degree, seriousness, and discharge condition can be found in the Supplementary Material (Table S2).

When comparing frequent admission diagnoses and symptoms with phenotype frequencies, we found an association of the CYP2C19 phenotype distribution with the admission diagnosis D62 (acute post-hemorrhagic anemia, *p* = 0.028), with a higher CYP2C19 activity (more RM and less NM, both *p* < 0.05) associated with that diagnosis. Comparisons of frequent admission diagnoses and symptoms with metabolic phenotypes can be found in the Supplementary Material (Table S3).

**Figure 1:**
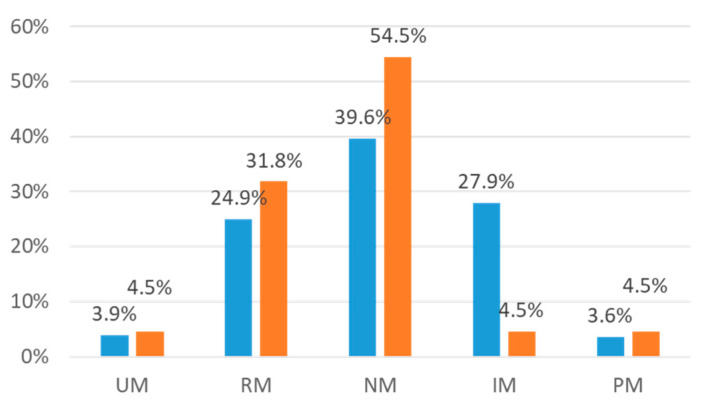
Differences in the metabolic phenotypes of CYP2C19 (*p* = 0.020) for not suspecting clopidogrel (blue columns) and suspecting clopidogrel (orange columns). UM: ultra-rapid metabolizer; RM: rapid metabolizer; NM: normal metabolizer; IM: intermediate metabolizer; PM: poor metabolizer.

Overall, suspecting clopidogrel for causing the ADR was associated with the CYP2C19 phenotype (*p* = 0.020). A higher activity of CYP2C19 with more clopidogrel-suspecting ADR cases was observed. The single difference for the phenotype groups differed significantly for IM (*p* < 0.05), but not for the other single phenotype groups, due to the small sample sizes. Those patients where clopidogrel was not suspected were 3.9% (*n* = 28) UM, 24.9% (*n* = 178) RM, 39.6% (*n* = 283) NM, 27.9% (*n* = 199) IM, and 3.6% (*n* = 26) PM. In contrast, those patients where clopidogrel was suspected were 4.5% (*n* = 2) UM, 31.8% (*n* = 14) RM, 54.5% (*n* = 24) NM, 4.5% (*n* = 2) IM, and 4.5% (*n* = 2) PM. Figure 1 shows the different frequencies of the CYP2C19 phenotypes and the suspected clopidogrel.

As the CYP2C19 phenotype was associated with suspecting clopidogrel ADRs, we included the CYP2C19_sum_ phenotype in two logistic regression models, to assess its influence together with the clinical factors to control for the effects of age and sex, and for the clinical multi-morbidity presented by the number of comorbidities and the number of drugs taken. The CYP2C19_sum_ phenotype summarizes low enzyme activity (IM and PM) and high enzyme activity (NM, RM, and UM). The results of the two models are pictured in Table 3.

In the logistic regression analysis, higher CYP2C19 activity (NM, RM, and UM) as presented by the CYP2C19_sum_ phenotype, together with age, showed an impact on suspecting clopidogrel for an ADR, leading to ED presentation in both models. The effect of the CYP2C19_sum_ phenotype was higher than the age effect and even increased slightly when including other clinical factors in the model (OR^1^ 4.85 (1.70–13.87) and OR^2^ 4.97 (1.73–14.27)).

Of those 45 ADR cases that were suspected to be caused by clopidogrel, 30 ADR (66.7%) were assessed as possible and 15 (33.3%) as probable relation to clopidogrel intake. Nearly all cases (44, 97.8% (1 case with missing information)) took a dose of 75 mg once daily. Information about indication for the intake of clopidogrel was available for 34 patients (75.6%), with chronic ischemic heart disease (I25: 22 (48.8%)) being the most common indication for treatment with clopidogrel. Of those 45 clopidogrel-suspecting ADR cases, 30 (66.7%) took additional acetylsalicylic acid (ASA), 8 (17.8%) took phenprocoumon, 6 (13.3%) took rivaroxaban, 3 (6.7%) took apixaban, 3 (6.7%) took ibuprofen, and 3 (6.7%) took prednisolone. None of those additional drugs was suspected to cause bleeding events. Of all clopidogrel-suspecting cases, 35 (77.8%) patients took a proton pump inhibitor (PPI), of which 4 (8.9%) took a PPI with strong inhibiting properties on CYP2C19 (esomeprazole, omeprazole). No other drug known as a CYP2C19 inhibitor (fluconazole, fluoxetine, fluvoxamine, ticlopedine, felbamate, voriconazole) was taken by the patients with suspected clopidogrel ADRs [26]. In contrast, one patient (2.2%) who was a CYP2C19 NM took a strong CYP2C19 inducer (rifampicin).

**Figure 2:**
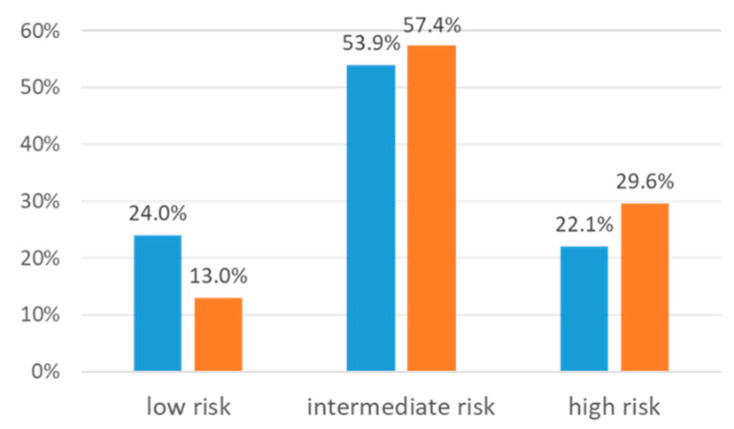
Differences in pharmacogenetic anticoagulant activity risk profiles for no suspected phenprocoumon (blue columns) and suspected phenprocoumon (orange columns), *p* = 0.052. Low risk was defined as being CYP2C9 NM and VKORC1 NC. Intermediate risk was defined as having either low CYP2C9 activity (intermediate metabolizer (IM) or poor metabolizer (PM)) or low VKORC1 activity (intermediate clotter (IC) or poor clotter (PC)). High-risk patients were those with a low CYP2C9 activity and a low VKORC1 activity.

The frequency distribution of the CYP2C9 phenotype did not differ significantly in the subgroup of the phenprocoumon-suspecting ADRs, compared to the rest. The same was true for the VKORC1 phenotypes. When combining the CYP2C9 and VKORC1 phenotypes into groups predicting anticoagulant activity, there was a trend for a higher frequency of the genotype groups predicting high anticoagulant activity in the ADR subgroup with a suspected phenprocoumon causative agent (*p* = 0.052), with no suspected phenprocoumon versus suspected phenprocoumon in low risk 24.0% (*n* = 161) versus 13.0% (*n* = 7), in intermediate risk 53.9% (*n* = 362) versus 57.4% (*n* = 31), and high risk profiles 22.1% (*n* = 148) versus 29.6% (*n* = 16), respectively. Figure 2 shows the frequency of the combined VCORC1 and CYP2C9 phenotype groups, sorted according to the anticoagulant activity, in comparison between the two groups—ADR cases without or with suspected phenprocoumon for being causative.

Even though only a statistical trend towards significance was observed, we included the summarized anticoagulant activity phenotype (anticoagulant activity_sum_) in two logistic regression models to assess its influence together with the clinical factors, to control for the effects of age and sex, and for the clinical multi-morbidity presented by the number of comorbidities and the number of drugs taken. The anticoagulant activity_sum_ phenotype summarizes the high and intermediate risk (low CYP2C9 activity (IM or PM) or/and low VKORC1 activity (IC or PC)) versus low risk (CYP2C9 NM and VKORC1 NC). The results of the two models are pictured in Table 4.

In the logistic regression analysis, the pharmacogenomic anticoagulant activity profile (intermediate and high risk) showed no effect on the suspected phenprocoumon for an ADR, when adjusting for clinical parameters, but age showed an effect in both models (OR^1^ and OR^2^, both 1.03 [1.01–1.05]).

Neither suspected metoprolol nor bisoprolol were found to be associated with CYP2D6 phenotype, when considered separately or when taken together. Likewise, suspected ibuprofen was not associated with the CYP2C9 phenotype.

## 3. Discussion

This study showed, for the first time, a clinical effect of the pharmacogenomic profiles for a large cohort of ADR cases treated in EDs. The PGx effect on the ADR cases was prominent at higher activity of CYP2C19, presenting with an ADR that was caused by the CYP2C19 substrate clopidogrel, but anticoagulant activity determined by the pharmacogenomics risk profiles of CYP2C9 combined with VKORC1 also showed an impact on the phenprocoumon-suspecting ADRs.

The finding that a higher activity of CYP2C19 was associated with a clopidogrel-suspecting ADR case causing the observed symptoms, was in line with other data. The oral antiplatelet agent clopidogrel was a prodrug, which was extensively metabolized into its active metabolite. Therefore, two sequential oxidative steps were needed, which were both performed by the polymorphic CYP2C19 [27]. Another study showed an increased risk of the bleeding events in the carriers of the CYP2C19*17 allele within 30 days after percutaneous coronary intervention (PCI) and concomitant intake of ASA [28]. A second study found an association of CYP2C19*17 on the bleeding events, up to one month after PCI [29]. Interestingly, in our study sample, nearly a quarter was at least heterozygous for the *17 allele (24.7% RM and 3.9% UM (homozygous)). Frequency distribution of the CYP2C19 phenotypes did not differ from an expected distribution in the European population, underlining that this population was not a special selection and pointing towards a general risk for patients that receive treatment with clopidogrel. Strikingly, the admission diagnosis of acute post-hemorrhagic anemia was likewise associated with a higher CYP2C19 enzyme activity, pointing to occult bleedings. However, sample sizes per suspected drug were too small for further analyses and the collection of more ADR cases together with biomaterial for genotyping is needed.

We do not know for how long the patients took clopidogrel prior to ED presentation in our dataset. However, most patients took clopidogrel for a chronic condition and in a third without any concomitant intake of ASA. This might be explained by an intolerance of ASA or more likely by a history of bleeding. It is often recommended to discontinue dual antiplatelet treatment (DAPT) with ASA and a P_2_Y_12_ inhibitor such as clopidogrel, after recurrent bleeding events [30]. Especially, discontinuation of clopidogrel was shown to be associated with coronary stent thrombosis leading to a discontinuation of ASA in the case of recurrent bleedings [31,32]. However, recurrent gastric ulcer bleeding was more common with clopidogrel compared to ASA single antiplatelet treatment (SAPT), in combination with a proton pump inhibitor [33]. Treatment decision about discontinuation of DAPT and the choice for a specific drug for SAPT remains a challenge in clinical reality. It might be that higher risk patients tend to be treated with clopidogrel more frequently, however outcomes seem comparable for SAPT with clopidogrel or ASA [34]. Something crucial to add is that PPIs that are usually given to prevent gastro intestinal bleeding can become ineffective on regular doses, due to reduced PPI plasma concentrations in CYP2C19 UM or RM [35]. Moreover, not only the genotype but also the drug–drug interactions can have an impact on the resulting phenotypes. Through CYP-enzyme inhibition, genotypic NM might eventually present phenotypically as PM [36]. The same way could lead to less UM with CYP-inhibiting co-medication than when extrapolated from a genotype. As in this study the presented patients were mainly multi-medicated, it is likely that the phenotypes would appear with less activity than expected, based on the genotype. However, in the case of clopidogrel PPIs, mostly known mild CYP2C19 inhibitors were taken by our population. In our dataset, clopidogrel was the most frequent P_2_Y_12_ inhibitor taken. Prasugrel, which is meant to be metabolized via alternative pathways and not via CYP2C19, was rarely taken by our population (*n* = 3) and was never suspected to cause an ADR. The prominent use of clopidogrel, compared to prasugrel, was in line with German prescription data [37]. Our results suggested that the decision about the right SAPT drug or the decision about the benefit–risk ratio of DAPT might differ, based on the pharmacogenomic profile. Interestingly, high CYP2C19 activity was associated with a clopidogrel-suspecting ADR, together with age, even when adjusting for other clinical parameters. Further studies on the benefit and risks of clopidogrel in SAPT or DAPT in the carriers of the CYP2C19*17 allele are needed, especially in the context of aging.

In our study, there was a statistical trend of pharmacogenomic high-risk profiles with anticoagulant activity being associated with phenprocoumon-suspecting ADRs. However, this effect was not present when adjusting for clinical parameters, while age reached significance. Although the sample size was too small to reach statistical significance, the finding was in line with the current knowledge of the PGx risk. The vitamin K antagonist (VKA) phenprocoumon is known to be metabolized by CYP2C9, amongst others, and decreasing function variants such as *2 or *3 were described to lead to a decreased metabolic capacity [38]. VKA such as phenprocoumon or warfarin, block a subunit of the enzyme vitamin K epoxidase reductase (VKOR), which leads to a lesser reactivation of vitamin K, resulting to a decrease in blood clotting activity. A polymorphism in this subunit of VKORC1 was known to lead to a higher sensitivity for VKA [39]. The CYP2C9 and the VKORC1 polymorphism might both influence dose stability and anticoagulation quality in phenprocoumon users [40]. While CYP2C9 is the major enzyme for the metabolism of warfarin, the VKA phenprocoumon used here was also metabolized by CYP3A4, which exposed it to many potential drug–drug or drug–food interactions. In this study sample, we did not control for food interactions or for drug–drug interactions that affected the phenprocoumon metabolism. Other studies showed that one or more genetic polymorphisms in those genes affect the occurrence of VKA-associated ADRs, such as phenprocoumon-associated bleeding [41,42,43]. However, anticoagulation with VKA was usually monitored by regular measurement of the international normalized ratio (INR), thereby providing a pharmacodynamic drug monitoring that offers the possibility for adjusting doses, based on an INR therapeutic range. This might explain why PGx-associated phenprocoumon-suspecting ADRs were not that prominent in our dataset. In comparison, the above-mentioned drug clopidogrel did not lead to symptoms and did not show any effect that can be easily measured.

The same explanation might be true for beta-blockers and ibuprofen. These drugs would usually lead to symptoms such as bradycardia and dizziness in the case of beta-blockers or nausea, and gastric pain in the case of ibuprofen, when extremely high individual doses are given. It is expected that these mild ADRs would lead to a discontinuation of treatment or a treatment modification [44]. Therefore, the pharmacogenomic variance would impact mostly those drugs that do not present with mild ADR, such as clopidogrel or phenprocoumon. In those drugs, we expect an individual difference in drug exposure, based on the pharmacogenomic profile and the altered drug metabolism that leads to occurrence of serious ADRs.

We assume our study population to be at least partially generalizable, as this was an older, multi-morbid, and multi-medicated cohort, which was in line with other studies of ADR in EDs [11,45]. Antipsychotics and antidepressants were not among the drugs most frequently suspected in absolute number in the present study. This might be due to the fact that no hospitals that enrolled these patients had a psychiatric ward. However, when analyzing the numbers of the suspected drugs in relation to its intake frequency, those drugs acting on the central nervous system, such as antipsychotics and antidepressants, were prominently associated with suspected ADRs, as shown in the larger dataset of the ADRED study [11]. There might be some limitations to the generalizability, as nearly all patients in our study sample were hospitalized. As the genetic testing needed informed consent, enrollment of patients was probably easier in hospitalized patients and those that stayed longer in hospital. Further, we expect the number of patients that died or became unconscious due to a serious ADR to be underrepresented in our study sample, due to the enrollment strategy for getting informed consent. Some findings need a more precise analysis that would need larger sample sizes, such as those comparisons of single metabolic phenotypes, single symptoms, and admission diagnoses. In addition, there might be another limitation in the interpretation of genotype results, as this is a constantly developing field and new findings always need to be considered. Therefore, the extrapolation of phenotypes from genotypes has limitations. Besides the above-mentioned phenoconversion due to drug–drug interactions, extrapolation of phenotypes from genotypes is challenging, especially in the case of CYP2D6. Discrepancies between phenotypes and genotypes exist [46] and multiplicated active CYP2D6 alleles only explain a fraction of the UM phenotype observed in Caucasian populations [47,48,49,50]. Additionally, the attribution of a CYP2D6 IM is based on a genotype composed normally of one reduced and one null allele [51,52,53], while the kinetic profiles in some IMs can be similar to those observed for PMs for some drugs [52]. Meanwhile, NMs cover large metabolic ratios and are presented by a variety of functional, reduced, or non-functional alleles. In the study presented, we used the most recent consensus statement for translating the CYP2D6 genotype into a phenotype [22].

In terms of the strength of our study, to the best of our knowledge this was the largest study population with serious dose-related ADRs and known pharmacogenomic profiles. While some reviews on this topic exist [9,54], we could show for the first time a pharmacogenomic effect on specific ADRs, leading to patient admission to the hospital ED.

## 4. Conclusions

We found a pharmacogenomic impact on serious ADRs leading to ED admissions. This impact might be highest in the drugs that cannot be easily monitored or that do not provoke mild ADR symptoms quickly, which itself could lead to treatment modifications and dose adaptation that prevent serious ADRs. Therefore, in particular patients that require the intake of such drugs or prodrugs, respectively, with known pharmacogenomic variability such as clopidogrel, codeine, PPIs or sulfonylureas, might benefit mostly from pharmacogenomic testing to prevent those serious ADRs and decrease the workload of EDs.

## Figures and Tables

**Table 1:** Characteristics of the study population. Continuous variables are presented as median (interquartile range). Categorical variables are presented in absolute numbers (percentages).

	Missing (*n*)	Cases, *n* = 776
Age (Years)	-	70 (56; 79)
Sex (Male)	-	445 (57.3%)
Ethnicity (Caucasian)	20 (2.6%)	752 (96.9%)
Drugs taken (Number)	-	8 (5; 11)
Drugs Suspected	-	1 (1; 2)
Comorbidities (Number)	-	6 (4; 10)
GFR (mL/min/1.73 m^2^)	356 (45.9%)	66.4 (44.1; 89.2)
Hospitalized	-	746 (96.1%)
Length of Stay (Days)	30 (3.9%)	7 (4; 10)
**Triage in the ED**	7 (0.9%)	
Red		104 (13.4%)
Orange		300 (38.7%)
Yellow		290 (37.4%)
Green/Blue		75 (9.7%)
**Admission diagnoses** (Number)	-	2 (1; 2)
K92		134 (17.3%)
N17		62 (8.0%)
E87		53 (6.8%)
D62		57 (7.3%)
R55		43 (5.5%)
**Symptoms** (PT, Number)	-	3 (2; 5)
General Physical Health Deterioration		178 (22.9%)
Dyspnea		144 (18.6%)
Dizziness		123 (15.9%)
Nausea		111 (14.3%)
Blood Stool		110 (14.2%)
**Seriousness on ED Arrival**	-	
Non serious harm		33 (4.3%)
Hospitalization required		669 (86.2%)
Life-threatening		74 (9.5%)
**Condition at Discharge**	14 (1.8%)	
Recovered		38 (4.9%)
Not Recovered		65 (8.4%)
Condition Improved		638 (82.2%)
Persistent Harm		3 (0.4%)
Death		18 (2.3%)
**CYP2D6 Phenotype**	36 (4.6%)	
UM		25 (3.2%)
NM		394 (50.8%)
IM		272 (35.1%)
PM		49 (6.3%)
**CYP2C19 Phenotype**	18 (2.3%)	
UM		30 (3.9%)
RM		192 (24.7%)
NM		307 (39.6%)
IM		201 (25.9%)
PM		28 (3.6%)
**CYP2C9 Phenotype**	14 (1.8%)	
NM		496 (63.9%)
IM		239 (30.8%)
PM		27 (3.5%)
**VKORC1 Phenotype**	51 (6.6%)	
NC		259 (33.4%)
IC		342 (44.1%)
PC		124 (16.0%)

Bold text presents subheadings; GFR: glomerular filtration rate; Triage in ED: red: resuscitation; orange: emergency; yellow: urgent; green/blue: semi-urgent/nonurgent; K92: Other diseases of digestive system; N17: Acute renal failure; E87: Other disorders of fluid, electrolyte and acid-base balance; D62: Acute post-hemorrhagic anemia; R55: syncope and collapse; PT: preferred term according to MedDRA; UM: ultra-rapid metabolizer; RM: rapid metabolizer; NM: normal metabolizer; IM: intermediate metabolizer; PM: poor metabolizer; NC: normal clotter; IC: intermediate clotter; and PC: poor clotter.

**Table 2:** Frequent admission diagnoses and symptoms per patients with suspected substance use.

Drug	Frequency Suspected, *n* (%)	Admission Diagnosis, *n* (%)	Symptom, *n* (%)
Acetylsalicylic Acid	96 (12.4)	K92, 60 (62.5)	Blood stool, 48 (50.0)
Phenprocoumon	60 (7.7)	K92, 21 (35.0)	Blood stool, 19 (31.7)
Prednisolone	53 (6.8)	K92, 8 (15.1)	Fever, 14 (26.4)
Clopidogrel	45 (5.8)	K92, 27 (60.0)	Blood stool, 23 (51.1)
Rivaroxaban	45 (5.8)	K92, 21 (46.7)	Blood stool, 17 (37.8)
Metoprolol	40 (4.1)	R55, 12 (30.0)	Dizziness, 16 (40.0)
Ibuprofen	38 (4.9)	K92, 10 (26.3)	Nausea, 9 (23.7)
Bisoprolol	32 (4.1)	I48, 9 (28.1)	Bradycardia, 9 (28.1)
Torasemide	30 (3.9)	E87, 12 (40.0)	Dizziness, 10 (33.3)
Apixaban	30 (3.9)	K92, 22 (73.3)	Blood stool, 16 (53.3)

K92: Other diseases of digestive system; R55: syncope and collapse; I48: atrial fibrillation and flutter; E87: Other disorders of fluid, electrolyte, and acid–base balance.

**Table 3:** Results of regression analyses for suspecting clopidogrel, based on the CYP2C19 enzyme activity.

	Age-Sex Adjusted OR (95% CI) ^1^	Multi-Adjusted OR (95% CI) ^2^
CYP2C19_sum_ (NM, RM, UM)	4.85 (1.70–13.87)	4.97 (1.73–14.27)
Age (Years)	1.05 (1.02–1.08)	1.05 (1.02–1.08)
Sex (Male)	1.05 (0.55–2.00)	1.13 (0.59–2.16)
Comorbidities (Number)	-	1.05 (0.98–1.12)
Drugs taken (Number)	-	1.06 (0.98–1.15)

^1^ Model including the CYP2C19_sum_ phenotype, age, and sex (R^2^ = 0.08). ^2^ Model including the CYP2C19_sum_ phenotype, age, sex, number of comorbidities, and number of drugs taken (R^2^ = 0.11).

**Table 4:** Results of the regression analyses for suspected phenprocoumon based on the pharmacogenomic anticoagulant activity-profile.

	Age-Sex Adjusted OR (95% CI) ^1^	Multi-Adjusted OR (95% CI) ^2^
Anticoagulant Activity_sum_ (Intermediate/High Risk)	1.90 (0.83–4.33)	1.90 (0.83–4.34)
Age (Years)	1.03(1.01–1.05)	1.03 (1.01–1.05)
Sex (Male)	0.71 (0.39–1.31)	0.71 (0.39–1.32)
Comorbidities (Number)	-	1.00 (0.92–1.08)
Drugs Taken (Number)	-	1.00 (0.93–1.08)

^1^ Model including anticoagulant activity_sum_ phenotype, age, and sex (R^2^ = 0.04). ^2^ Model including anticoagulant activity_sum_ phenotype, age, sex, number of comorbidities, and number of drugs taken (R^2^ = 0.04).

## Supplementary Materials

The following are available online at https://www.mdpi.com/2077-0383/9/6/1801/s1. Table S1. Definition of phenotypes in the study population. Table S2. Comparison of triage (A), ADR seriousness (B), and condition at discharge (C) with metabolic phenotypes. Frequencies given in absolute numbers (percentages). Table S3. Comparisons with admission diagnoses (A) and symptoms (B) with metabolic phenotypes. Frequencies given in absolute numbers (percentages), *p*-values for the given phenotype distribution.

## References

[1] Schurig A.M., Bohme M., Just K.S., Scholl C., Dormann H., Plank-Kiegele B., Seufferlein T., Graff I., Schwab M., Stingl J.C. (2018). Adverse Drug Reactions (ADR) and Emergencies. Deutsches Arzteblatt Int..

[2] Pirmohamed M., James S., Meakin S., Green C., Scott A.K., Walley T.J., Farrar K., Park B.K., Breckenridge A.M. (2004). Adverse drug reactions as cause of admission to hospital: Prospective analysis of 18 820 patients. BMJ.

[3] Lazarou J., Pomeranz B.H., Corey P.N. (1998). Incidence of adverse drug reactions in hospitalized patients: A meta-analysis of prospective studies. JAMA.

[4] Just K.S., Dormann H., Schurig M., Bohme M., Steffens M., Plank-Kiegele B., Ettrich K., Seufferlein T., Graff I., Igel S. (2020). The phenotype of adverse drug effects: Do emergency visits due to adverse drug reactions look different in older people?—Results from the ADRED study. Br. J. Clin. Pharmacol..

[5] Edwards I.R., Aronson J.K. (2000). Adverse drug reactions: Definitions, diagnosis, and management. Lancet.

[6] Evans W.E., Relling M.V. (1999). Pharmacogenomics: Translating functional genomics into rational therapeutics. Science.

[7] Hamburg M.A., Collins F.S. (2010). The path to personalized medicine. N. Engl. J. Med..

[8] Plumpton C.O., Roberts D., Pirmohamed M., Hughes D.A. (2016). A systematic review of economic evaluations of pharmacogenetic testing for prevention of adverse drug reactions. Pharmacoeconomics.

[9] Phillips K.A., Veenstra D.L., Oren E., Lee J.K., Sadee W. (2001). Potential role of pharmacogenomics in reducing adverse drug reactions: A systematic review. JAMA.

[10] Stingl J., Brockmöller J., Viviani R. (2013). Genetic variability of drug-metabolizing enzymes: The dual impact on psychiatric therapy and regulation of brain function. Mol. Psychiatry.

[11] Just K.S., Dormann H., Böhme M., Schurig M., Schneider K.L., Steffens M., Dunow S., Plank-Kiegele B., Ettrich K., Seufferlein T. (2020). Personalising drug safety—Results from the multi-centre prospective observational study on Adverse Drug Reactions in Emergency Departments (ADRED). Eur. J. Clin. Pharmacol..

[12] Eichelbaum M., Ingelman-Sundberg M., Evans W.E. (2006). Pharmacogenomics and individualized drug therapy. Annu. Rev. Med..

[13] Leckband S., Kelsoe J., Dunnenberger H., George A., Tran E., Berger R., Müller D., Whirl-Carrillo M., Caudle K., Pirmohamed M. (2013). Clinical Pharmacogenetics Implementation Consortium guidelines for HLA-B genotype and carbamazepine dosing. Clin. Pharmacol. Ther..

[14] Martin M., Klein T., Dong B., Pirmohamed M., Haas D., Kroetz D. (2012). Clinical pharmacogenetics implementation consortium guidelines for HLA-B genotype and abacavir dosing. Clin. Pharmacol. Ther..

[15] Elliott L.S., Henderson J.C., Neradilek M.B., Moyer N.A., Ashcraft K.C., Thirumaran R.K. (2017). Clinical impact of pharmacogenetic profiling with a clinical decision support tool in polypharmacy home health patients: A prospective pilot randomized controlled trial. PLoS ONE.

[16] Johnson J.A., Caudle K.E., Gong L., Whirl-Carrillo M., Stein C.M., Scott S.A., Lee M.T., Gage B.F., Kimmel S.E., Perera M.A. (2017). Clinical Pharmacogenetics Implementation Consortium (CPIC) Guideline for Pharmacogenetics-Guided Warfarin Dosing: 2017 Update. Clin. Pharmacol. Ther..

[17] Crews K.R., Gaedigk A., Dunnenberger H.M., Leeder J.S., Klein T.E., Caudle K.E., Haidar C.E., Shen D.D., Callaghan J.T., Sadhasivam S. (2014). Clinical Pharmacogenetics Implementation Consortium guidelines for cytochrome P450 2D6 genotype and codeine therapy: 2014 update. Clin. Pharmacol. Ther..

[18] Van der Wouden C.H., Bohringer S., Cecchin E., Cheung K.C., Davila-Fajardo C.L., Deneer V.H.M., Dolzan V., Ingelman-Sundberg M., Jonsson S., Karlsson M.O. (2020). Generating evidence for precision medicine: Considerations made by the Ubiquitous Pharmacogenomics Consortium when designing and operationalizing the PREPARE study. Pharmacogenet. Genom..

[19] Wood K. (1994). The medical dictionary for drug regulatory affairs (MEDDRA) project. Pharmacoepidemiol. Drug Saf..

[20] WHO International Classification of Diseases, Version 10. http://www.who.int/classifications/icd/icd10updates/en/.

[21] Matsushita K., Mahmoodi B.K., Woodward M., Emberson J.R., Jafar T.H., Jee S.H., Polkinghorne K.R., Shankar A., Smith D.H., Tonelli M. (2012). Comparison of risk prediction using the CKD-EPI equation and the MDRD study equation for estimated glomerular filtration rate. JAMA.

[22] Caudle K.E., Sangkuhl K., Whirl-Carrillo M., Swen J.J., Haidar C.E., Klein T.E., Gammal R.S., Relling M.V., Scott S.A., Hertz D.L. (2020). Standardizing CYP2D6 Genotype to Phenotype Translation: Consensus Recommendations from the Clinical Pharmacogenetics Implementation Consortium and Dutch Pharmacogenetics Working Group. Clin. Transl. Sci..

[23] PharmGKB PGx Gene-specific Information Tables. https://www.pharmgkb.org/page/pgxGeneRef.

[24] GraphPad QuickCalcs—Compare Observed and Expected Frequencies. https://www.graphpad.com/quickcalcs/chisquared1.cfm.

[25] Whirl-Carrillo M., McDonagh E.M., Hebert J., Gong L., Sangkuhl K., Thorn C., Altman R.B., Klein T.E. (2012). Pharmacogenomics knowledge for personalized medicine. Clin. Pharmacol. Ther..

[26] FDA Drug Development and Drug Interactions: Table of Substrates, Inhibitors, and Inducers. https://www.fda.gov/drugs/drug-interactions-labeling/drug-development-and-drug-interactions-table-substrates-inhibitors-and-inducers.

[27] Kazui M., Nishiya Y., Ishizuka T., Hagihara K., Farid N.A., Okazaki O., Ikeda T., Kurihara A. (2010). Identification of the human cytochrome P450 enzymes involved in the two oxidative steps in the bioactivation of clopidogrel to its pharmacologically active metabolite. Drug Metab. Dispos..

[28] Sibbing D., Koch W., Gebhard D., Schuster T., Braun S., Stegherr J., Morath T., Schomig A., von Beckerath N., Kastrati A. (2010). Cytochrome 2C19*17 allelic variant, platelet aggregation, bleeding events, and stent thrombosis in clopidogrel-treated patients with coronary stent placement. Circulation.

[29] Campo G., Parrinello G., Ferraresi P., Lunghi B., Tebaldi M., Miccoli M., Marchesini J., Bernardi F., Ferrari R., Valgimigli M. (2011). Prospective evaluation of on-clopidogrel platelet reactivity over time in patients treated with percutaneous coronary intervention relationship with gene polymorphisms and clinical outcome. J. Am. Coll. Cardiol..

[30] Halvorsen S., Storey R.F., Rocca B., Sibbing D., Ten Berg J., Grove E.L., Weiss T.W., Collet J.P., Andreotti F., Gulba D.C. (2017). Management of antithrombotic therapy after bleeding in patients with coronary artery disease and/or atrial fibrillation: Expert consensus paper of the European Society of Cardiology Working Group on Thrombosis. Eur. Heart J..

[31] Park D.W., Park S.W., Park K.H., Lee B.K., Kim Y.H., Lee C.W., Hong M.K., Kim J.J., Park S.J. (2006). Frequency of and risk factors for stent thrombosis after drug-eluting stent implantation during long-term follow-up. Am. J. Cardiol..

[32] Van Werkum J.W., Heestermans A.A., Zomer A.C., Kelder J.C., Suttorp M.J., Rensing B.J., Koolen J.J., Brueren B.R., Dambrink J.H., Hautvast R.W. (2009). Predictors of coronary stent thrombosis: The Dutch Stent Thrombosis Registry. J. Am. Coll. Cardiol..

[33] Chan F.K., Ching J.Y., Hung L.C., Wong V.W., Leung V.K., Kung N.N., Hui A.J., Wu J.C., Leung W.K., Lee V.W. (2005). Clopidogrel versus aspirin and esomeprazole to prevent recurrent ulcer bleeding. N. Engl. J. Med..

[34] Lemesle G., Schurtz G., Meurice T., Tricot O., Lemaire N., Caudmont S., Philias A., Ketelers R., Lamblin N., Bauters C. (2016). Clopidogrel Use as Single Antiplatelet Therapy in Outpatients with Stable Coronary Artery Disease: Prevalence, Correlates and Association with Prognosis (from the CORONOR Study). Cardiology.

[35] Swen J.J., Nijenhuis M., de Boer A., Grandia L., Maitland-van der Zee A.H., Mulder H., Rongen G.A., van Schaik R.H., Schalekamp T., Touw D.J. (2011). Pharmacogenetics: From bench to byte—An update of guidelines. Clin. Pharmacol. Ther..

[36] Shah R.R., Smith R.L. (2015). Addressing phenoconversion: The Achilles’ heel of personalized medicine. Br. J. Clin. Pharmacol..

[37] Schwabe U., Paffrath D., Ludwig W., Klauber J. (2017). Arzneiverordnungs-Report 2017.

[38] Kirchheiner J., Ufer M., Walter E.C., Kammerer B., Kahlich R., Meisel C., Schwab M., Gleiter C.H., Rane A., Roots I. (2004). Effects of CYP2C9 polymorphisms on the pharmacokinetics of R- and S-phenprocoumon in healthy volunteers. Pharmacogenetics.

[39] Reitsma P.H., van der Heijden J.F., Groot A.P., Rosendaal F.R., Buller H.R. (2005). A C1173T dimorphism in the VKORC1 gene determines coumarin sensitivity and bleeding risk. PLoS Med..

[40] Schneider K.L., Kunst M., Leuchs A.K., Bohme M., Weckbecker K., Kastenmuller K., Bleckwenn M., Holdenrieder S., Coch C., Hartmann G. (2019). Phenprocoumon Dose Requirements, Dose Stability and Time in Therapeutic Range in Elderly Patients with CYP2C9 and VKORC1 Polymorphisms. Front. Pharmacol..

[41] Misasi S., Martini G., Paoletti O., Calza S., Scovoli G., Marengoni A., Testa S., Caimi L., Marchina E. (2016). VKORC1 and CYP2C9 polymorphisms related to adverse events in case-control cohort of anticoagulated patients. Medicine.

[42] Bryk A.H., Wypasek E., Plens K., Awsiuk M., Undas A. (2018). Bleeding predictors in patients following venous thromboembolism treated with vitamin K antagonists: Association with increased number of single nucleotide polymorphisms. Vascul. Pharmacol..

[43] Sridharan K., Modi T., Bendkhale S., Kulkarni D., Gogtay N.J., Thatte U.M. (2016). Association of Genetic Polymorphisms of CYP2C9 and VKORC1 with Bleeding Following Warfarin: A Case-Control Study. Curr. Clin. Pharmacol..

[44] Leporini C., De Sarro G., Russo E. (2014). Adherence to therapy and adverse drug reactions: Is there a link?. Expert Opin. Drug Saf..

[45] Budnitz D.S., Lovegrove M.C., Shehab N., Richards C.L. (2011). Emergency hospitalizations for adverse drug events in older Americans. N. Engl. J. Med..

[46] LLerena A., Naranjo M.E., Rodrigues-Soares F., Penas L.E.M., Fariñas H., Tarazona-Santos E. (2014). Interethnic variability of CYP2D6 alleles and of predicted and measured metabolic phenotypes across world populations. Expert Opin. Drug Metab. Toxicol..

[47] Dahl M.L., Johansson I., Bertilsson L., Ingelman-Sundberg M., Sjöqvist F. (1995). Ultrarapid hydroxylation of debrisoquine in a Swedish population. Analysis of the molecular genetic basis. J. Pharmacol. Exp. Ther..

[48] Sachse C., Brockmöller J., Bauer S., Roots I. (1997). Cytochrome P450 2D6 variants in a Caucasian population: Allele frequencies and phenotypic consequences. Am. J. Hum. Genet..

[49] Bathum L., Johansson I., Ingelman-Sundberg M., Hørder M., Brøsen K. (1998). Ultrarapid metabolism of sparteine: Frequency of alleles with duplicated CYP2D6 genes in a Danish population as determined by restriction fragment length polymorphism and long polymerase chain reaction. Pharmacogenetics.

[50] Løvlie R., Daly A.K., Matre G.E., Molven A., Steen V.M. (2001). Polymorphisms in CYP2D6 duplication-negative individuals with the ultrarapid metabolizer phenotype: A role for the CYP2D6*35 allele in ultrarapid metabolism?. Pharmacogenetics.

[51] Gaedigk A., Simon S.D., Pearce R.E., Bradford L.D., Kennedy M.J., Leeder J.S. (2008). The CYP2D6 activity score: Translating genotype information into a qualitative measure of phenotype. Clin. Pharmacol. Ther..

[52] Zanger U.M., Raimundo S., Eichelbaum M. (2004). Cytochrome P450 2D6: Overview and update on pharmacology, genetics, biochemistry. Naunyn. Schmiedebergs Arch. Pharmacol..

[53] Zanger U.M., Schwab M. (2013). Cytochrome P450 enzymes in drug metabolism: Regulation of gene expression, enzyme activities, and impact of genetic variation. Pharmacol. Ther..

[54] Meyer U.A. (2000). Pharmacogenetics and adverse drug reactions. Lancet.

