# Supplementary material for: Adverse Drug Reactions in the Emergency Department: Is There a Role for Pharmacogenomic Profiles at Risk?—Results from the ADRED Study
Source: J Clin Med. 2020 Jun 9;9(6):1801. doi: 10.3390/jcm9061801 (PMC7355597; doi:10.3390/jcm9061801)
Supplement: Supplementary file 1 [file jcm-09-01801-s001.pdf]

Supplementary material

Adverse drug reactions in the emergency department: Is there a role for pharmacogenomic profiles at risk? – Results from the ADRED study

Katja S Just (ORCID 0000-0002-6782-8078)[1], Harald Dormann[2], Marlen Schurig[3], Miriam Böhme[3], Jochen Fracowiak[3], Michael Steffens[3], Catharina Scholl[3], Thomas Seufferlein[4], Ingo Gräff[5], Matthias Schwab[6,7,8], Julia C Stingl[1*]

1 Institute of Clinical Pharmacology, University Hospital of RWTH Aachen, Aachen, Germany; kjust@ukaachen.de; jstingl@ukaachen.de
2 Central Emergency Department, Hospital Fürth, Fürth, Germany; Harald.Dormann@klinikum-fuerth.de
3 Research Department, Federal Institute for Drugs and Medical Devices, Bonn, Germany; marlen.schurig@bfarm.de; Miriam.boehme@bfarm.de; Michael.steffens@bfarm.de; Catharina.scholl@bfarm-research.de
4 Internal Medicine Emergency Department, Ulm University Medical Centre, Ulm, Germany; Thomas.Seufferlein@uniklinik-ulm.de
5 Interdisciplinary Emergency Department (INZ), University Hospital of Bonn, Bonn, Germany; ingo.graeff@ukb.uni-bonn.de
6 Dr. Margarete Fischer-Bosch-Institute of Clinical Pharmacology, Stuttgart, Germany; Matthias.Schwab@ikp-stuttgart.de
7 Department of Clinical Pharmacology, University of Tuebingen, Tuebingen, Germany; Matthias.Schwab@ikp-stuttgart.de
8 Department of Pharmacy and Biochemistry, University of Tuebingen, Tuebingen, Germany; Matthias.Schwab@ikp-stuttgart.de
* Correspondence: jstingl@ukaachen.de; Tel.: +49 241 8089130

Table S1. Definition of phenotypes in the study population.

Phenotype	Diplotypes
**CYP2C19**	
UM	*17/*17
RM	*1/*17
NM	*1/*1
IM	*1/*2; *1/*3; *1/*4A; *1/*8; *2/*17; *8/*17
PM	*2/*2; *2/*8
**CYP2C9**	
NM	*1/*1
IM	*1/*2; *1/*3; *1/*12
PM	*2/*2; *2/*3; *3/*3; *2/*11; *3/*11
**CYP2D6**	
UM (each CNV ≥3)	*1/*1; *1/*2; *2/*2; *1/*41; *2/*41; *39/*39
NM	*1/*1; *1/*2; *1/*4[2]; *1/*9; *1/*10; *1/*17; *1/*39; *1/*41; *1/*91; *1/*119; *2/*2; *2/*4[2]; *2/*10; *2/*9; *2/*41;*9/*41[2]; *39/*39; *39/*41

IM	*1/*1[1]; *1/*2[1]; *1/*3; *1/*4; *1/*5; *1/*6; *1/*9; *1/*10[1]; *1/*39[1]; *1/*41[1]; *2/*2[1]; *2/*3; *2/*4; *2/*5; *2/*6; *2/*9; *2/*41[1]; *3/*41; *4/*9; *4/*10; *4/*39; *4/*41; *4/*132; *5/*9; *5/*10; *5/*41; *5/*119; *6/*41; *9/*10; *9/*41; *10/*41; *41/*41; *119/*119
PM	*1/*3[1]; *1/*4[1]; *3/*4; *3/*5; *4/*4; *4/*5; *4/*6; *4/*10[1]; *4/*132[1]; *5/*5; *5/*6
**VKORC1**	
NC	*1/*1
IC	*1/*2
PC	*2/*2

UM: ultra-rapid metabolizer; RM: rapid metabolizer; NM: normal metabolizer; IM: intermediate metabolizer; PM: poor metabolizer; NC: normal clotter; IC: intermediate clotter; PC: poor clotter. CNV: copy number variation; [1]CNV=1 + non-functional hybrid allele; [2]CNV≥3.
Diplotypes for CYP2D6 were interpreted taking the copy number and the hybrid alleles into account in order to assess the number of functional alleles and to determine the activity score. Based on the activity score, diplotypes were classified into the following metabolic classes according to the CPIC interpretation notes: PM, IM, NM, and UM. The absence of any copy number signified to us the presence of a gene deletion (*5-allele).

Table S2. Comparison of triage (A), ADR seriousness (B), and condition at discharge (C) with metabolic phenotypes. Frequencies given in absolute numbers (percentages).

Phenotype	Red	Orange	Yellow	Green/ Blue	p-value
**CYP2D6**					0.476
UM	2 (2.0)	10 (3.4)	11 (4.1)	2 (2.7)	
NM	52 (51.5)	157 (54.0)	135 (50.6)	47 (62.7)	
IM	36 (35.6)	104 (35.7)	107 (40.1)	22 (29.3)	
PM	11 (10.9)	20 (6.9)	14 (5.2)	4 (5.3)	
**CYP2C19**					0.548
UM	5 (4.9)	13 (4.4)	11 (3.9)	1 (1.3)	
RM	26 (25.5)	83 (28.0)	64 (22.9)	18 (24.0)	
NM	38 (37.3)	109 (36.8)	120 (43.0)	38 (50.7)	
IM	31 (30.4)	82 (27.7)	71 (25.4)	15 (20.0)	
PM	2 (2.0)	9 (3.0)	13 (4.7)	3 (4.0)	
**CYP2C9**					0.436
NM	62 (60.8)	190 (64.0)	190 (67.6)	49 (65.3)	
IM	34 (33.3)	96 (32.3)	81 (28.8)	26 (34.7)	
PM	6 (5.9)	11 (3.7)	10 (3.6)	-	
**VKORC1**					0.427
NC	33 (33.7)	104 (36.6)	99 (37.1)	21 (29.6)	
IC	47 (48.0)	128 (45.1)	132 (49.4)	33 (46.5)	
PC	18 (18.4)	52 (18.3)	36 (13.5)	17 (23.9)	

Phenotype	Non-serious harm	Hospitalization required	Life-threatening	p-value
**CYP2D6**				0.181
UM	-	24 (3.8)	1 (1.4)	
NM	19 (57.6)	339 (53.4)	36 (50.0)	
IM	14 (42.4)	232 (36.5)	26 (36.1)	
PM	-	40 (6.3)	9 (12.5)	

**CYP2C19**				0.781
UM	1 (3.0)	27 (4.1)	2 (2.8)	
RM	10 (30.3)	165 (25.3)	17 (23.6)	
NM	14 (42.4)	268 (41.0)	25 (34.7)	
IM	8 (24.2)	169 (25.9)	24 (33.3)	
PM	-	24 (3.7)	4 (5.6)	
**CYP2C9**				0.708
NM	23 (69.7)	427 (65.0)	46 (63.9)	
IM	10 (30.3)	207 (31.5)	22 (30.6)	
PM	-	23 (3.5)	4 (5.6)	
**VKORC1**				0.523
NC	9 (28.1)	230 (36.9)	20 (29.0)	
IC	16 (50.0)	288 (46.2)	38 (55.1)	
PC	7 (21.9)	106 (17.0)	11 (15.9)	

Phenotype	Recovered	Not recovered	Condition improved	Persistent harm	Death	p-value
**CYP2D6**						0.902
UM	-	2 (3.2)	22 (3.6)	-	-	
NM	20 (52.6)	40 (63.5)	319 (52.6)	2 (66.7)	8 (47.1)	
IM	15 (39.5)	18 (28.6)	225 (37.1)	1 (33.3)	7 (41.2)	
PM	3 (7.9)	3 (4.8)	40 (6.6)	-	2 (11.8)	
**CYP2C19**						0.297
UM	2 (5.3)	2 (3.2)	26 (4.2)	-	-	
RM	9 (23.7)	18 (28.6)	154 (24.7)	-	6 (33.3)	
NM	20 (52.6)	23 (36.5)	256 (41.1)	3 (100.0)	2 (11.1)	
IM	6 (15.8)	18 (28.6)	164 (26.3)	-	8 (44.4)	
PM	1 (2.6)	2 (3.2)	23 (3.7)	-	2 (11.1)	
**CYP2C9**						0.860
NM	22 (57.9)	41 (64.1)	412 (65.9)	2 (66.7)	12 (66.7)	
IM	13 (34.2)	22 (34.4)	192 (30.7)	1 (33.3)	5 (27.8)	
PM	3 (7.9)	1 (1.6)	21 (3.4)	-	1 (5.6)	
**VKORC1**						0.061
NC	17 (44.7)	15 (24.6)	220 (36.9)	2 (66.7)	1 (6.7)	
IC	13 (34.2)	35 (57.4)	276 (46.3)	-	11 (73.3)	
PC	8 (21.1)	11 (18.0)	100 (16.8)	1 (33.3)	3 (20.0)	

Compared with chi-squared test.

Table S3. Comparisons with admission diagnoses (A) and symptoms (B) with metabolic phenotypes. Frequencies given in absolute numbers (percentages), p-values for phenotype distribution given.

Phenotype	K92 (yes/no)	N17 (yes/no)	E87 (yes/no)	D62 (yes/no)	R55 (yes/no)
**CYP2D6**	p=0.528	p=0.456	p=0.102	p=0.537	p=0.465
UM	4 (3.1)/ 21 (3.4)	2 (3.3)/ 23 (3.4)	0 (0)/ 25 (3.6)	2 (3.6)/ 23 (3.4)	0 (0)/ 25 (3.6)
NM	61 (47.7)/ 333 (54.4)	34 (56.7)/ 360 (52.9)	27 (52.9)/ 367 (53.3)	26 (46.4)/ 386 (53.8)	26 (61.9)/ 386 (52.7)
IM	54 (42.2)/ 218 (35.6)	23 (38.3)/ 249 (36.6)	17 (33.3)/ 255 (37.0)	22 (39.3)/ 250 (36.5)	13 (31.0)/ 259 (37.1)
PM	9 (7.0)/ 40 (6.5)	1 (1.7)/ 48 (7.1)	7 (13.7)/ 42 (6.1)	6 (10.7)/ 43 (6.3)	3 (7.1)/ 46 (6.6)

**CYP2C19**	p=0.615	p=0.754	p=0.999	**p=0.028**	p=0.608
UM	3 (2.3)/ 27 (4.3)	2 (3.2)/ 28 (4.0)	2 (3.8)/ 28 (4.0)	4 (7.0)/ 26 (3.7)	0 (0)/ 30 (4.2)
RM	35 (26.7)/ 157 (25.0)	14 (22.6)/ 178 (25.6)	13 (25.0)/ 179 (25.4)	23 (40.4)/ 169 (24.1)	9 (20.9)/ 183 (25.6)
NM	50 (38.2)/ 257 (41.0)	23 (37.1)/ 284 (40.8)	22 (42.3)/ 285 (40.4)	14 (24.6)/ 293 (41.8)	20 (46.5)/ 287 (40.1)
IM	36 (27.5)/ 165 (26.3)	21 (33.9)/ 180 (25.9)	13 (25.0)/ 188 (26.6)	14 (24.6)/ 187 (26.7)	12 (27.9)/ 189 (26.4)
PM	7 (5.3)/ 21 (3.3)	2 (3.2)/ 26 (3.7)	2 (3.8)/ 26 (3.7)	2 (3.5)/ 26 (3.7)	2 (4.7)/ 26 (3.6)
**CYP2C9**	p=0.488	p=0.672	p=0.583	p=0.293	p=0.430
NM	80 (60.6)/ 416 (66.0)	42 (67.7)/ 454 (64.9)	37 (71.2)/ 459 (64.6)	37 (64.9)/ 459 (65.1)	28 (65.1)/ 468 (65.1)
IM	47 (35.6)/ 192 (30.5)	19 (30.6)/ 220 (31.4)	14 (26.9)/ 225 (31.7)	20 (35.1)/ 219 (31.1)	12 (27.9)/ 227 (31.6)
PM	5 (3.8)/ 22 (3.5)	1 (1.6)/ 26 (3.7)	1 (1.9)/ 26 (3.7)	0 (0)/ 27 (3.8)	3 (7.0)/ 24 (3.3)
**VKORC1**	p=0.321	p=0.224	p=0.532	p=0.176	p=0.396
NC	39 (30.5)/ 220 (36.9)	26 (43.3)/ 233 (35.0)	15 (30.6)/ 244 (36.1)	21 (37.5)/ 238 (35.6)	17 (40.5)/ 242 (35.4)
IC	63 (49.2)/ 279 (46.7)	28 (46.7)/ 314 (47.2)	23 (46.9)/ 319 (47.2)	21 (37.5)/ 321 (48.0)	21 (50.0)/ 321 (47.0)
PC	26 (20.3)/ 98 (16.4)	6 (10.0)/ 118 (17.7)	11 (22.4)/ 113 (16.7)	14 (25.0)/ 110 (16.4)	4 (9.5)/ 120 (17.6)

**Phenotype**	**General physical health deterioration (yes/ no)**	**Dyspnea (yes/ no)**	**Dizziness (yes/ no)**	**Nausea (yes/ no)**	**Blood stool (yes/ no)**
**CYP2D6**	p=0.628	p=0.247	p=0.396	p=0.808	p=0.961
UM	4 (2.3)/ 21 (3.7)	3 (2.2)/ 22 (3.6)	1 (0.8)/ 24 (3.9)	4 (3.9)/ 21 (3.3)	3 (2.9)/ 22 (3.5)
NM	95 (54.6)/ 299 (52.8)	72 (52.6)/ 322 (53.4)	67 (56.3)/ 229 (52.7)	58 (56.3)/ 336 (52.7)	56 (53.3)/ 338 (53.2)
IM	61 (35.1)/ 211 (37.3)	48 (35.0)/ 224 (37.1)	43 (36.1)/ 229 (36.9)	36 (35.0)/ 236 (37.0)	38 (36.2)/ 234 (36.9)
PM	14 (8.0)/ 35 (6.2)	14 (10.2)/ 35 (5.8)	8 (6.7)/ 41 (6.6)	5 (4.9)/ 44 (6.9)	8 (7.6)/ 41 (6.5)
**CYP2C19**	p=0.634	p=0.791	p=0.153	p=0.738	p=0.442
UM	7 (4.0)/ 23 (4.0)	5 (3.6)/ 25 (4.0)	4 (3.3)/ 26 (4.1)	4 (3.8)/ 26 (4.0)	4 (3.7)/ 26 (4.0)
RM	46 (26.0)/ 146 (25.1)	35 (25.0)/ 157 (25.4)	32 (26.4)/ 160 (25.1)	26 (24.5)/ 166 (25.5)	33 (30.8)/ 159 (24.4)
NM	64 (36.2)/ 243 (41.8)	52 (37.1)/ 255 (41.3)	59 (48.8)/ 248 (38.9)	49 (46.2)/ 258 (39.6)	38 (35.5)/ 269 (41.3)
IM	54 (30.5)/ 147 (25.3)	43 (30.7)/ 158 (25.6)	24 (19.8)/ 177 (27.8)	24 (22.6)/ 177 (27.1)	26 (24.3)/ 175 (26.9)
PM	6 (3.4)/ 22 (3.8)	5 (3.6)/ 23 (3.7)	32 (26.4)/ 160 (25.1)	3 (2.8)/ 25 (3.8)	6 (5.6)/ 22 (3.4)
**CYP2C9**	p=0.473	p=0.796	p=0.601	p=0.978	p=0.727

NM	119 (67.2)/ 377 (64.4.)	87 (62.1)/ 409 (65.8)	75 (61.5)/ 421 (65.8)	71 (65.7)/ 425 (65.0)	67 (62.6)/ 429 (65.5)
IM	50 (28.2)/ 189 (32.3)	48 (34.3)/ 191 (30.7)	43 (35.2)/ 196 (30.6)	33 (30.6)/ 206 (31.5)	35 (32.7)/ 204 (31.3)
PM	8 (4.5)/ 19 (3.2)	5 (3.6)/ 22 (3.5)	4 (3.3)/ 23 (3.6)	4 (3.7)/ 23 (3.5)	5 (4.7)/ 22 (3.4)
**VKORC1**	p=0.289	p=0.184	p=0.728	p=0.816	p=0.696
NC	59 (34.9)/ 200 (36.0)	39 (28.9)/ 220 (37.3)	38 (33.9)/ 221 (36.1)	35 (34.3)/ 224 (36.0)	35 (33.7)/ 224 (36.1)
IC	87 (51.5)/ 255 (45.9)	70(51.9)/ 272 (46.1)	52 (46.4)/ 290 (47.3)	51 (50.0)/ 291 (46.7)	53 (51.0)/ 289 (46.5)
PC	23 (13.6)/ 101 (18.2)	26 (19.3)/ 98 (16.6)	22 (19.6)/ 102 (16.6)	16 (15.7)/ 108 (17.3)	16 (15.4)/ 108 (17.4)

Compared with chi-squared test.
